# *Capripoxviruses*: Exploring the genetic relatedness between field and vaccine strains from Egypt

**DOI:** 10.14202/vetworld.2019.1924-1930

**Published:** 2019-12-11

**Authors:** Sherin Reda Rouby, Abdel-Hamid Bazid, Momtaz Wasfy, Magdy El-Sayed

**Affiliations:** 1 Department of Veterinary Medicine, Faculty of Veterinary Medicine, Beni-Suef University, Beni-Suef 62511, Egypt; 2 Department of Virology, Faculty of Veterinary Medicine, University of Sadat City, Sadat City, Menoufia 32897, Egypt; 3 Department of Research and Development, Middle East for Veterinary vaccine Company (ME-VAC), Second Industrial Area, El-Salhya El-Gedida, Sharqia, Egypt; 4 Department of Internal Medicine and Infectious Diseases, Faculty of Veterinary Medicine, Cairo University, Giza Governorate 12613, Egypt

**Keywords:** *Capripoxvirus*, G protein-coupled receptors gene, phylogenetic, Romanian

## Abstract

**Background and Aim:**

Lumpy skin disease (LSD) and sheep pox are economically important *Capripoxvirus*-induced diseases of cattle and sheep, respectively. Despite the extensive vaccination program adopted by Egyptian veterinary authorities, LSD and sheep pox are still prevalent and spread throughout the whole country. The current study was designed for molecular characterization and phylogenetic analysis of LSD virus (LSDV) and *Sheep pox virus* (SPPV) recovered from field cases in Egypt along with vaccinal strains to assess their genetic relatedness.

**Materials and Methods:**

Skin biopsies were collected from naturally infected cases of LSD in Ismailia (n=3 farms) and Beni-Suef (n=2 farms) Governorates and sheep pox in Beni-Suef (n=1 flock). Virus isolation was carried out on primary ovine fetal kidney and heart cell cultures. DNA was extracted from infected materials (skin lesions, infected cell cultures) as well as LSDV Neethling vaccine strain and Romanian SPPV vaccine strain. Polymerase chain reaction was performed using oligonucleotide primers targeting the entire open reading frame of G protein-coupled receptors (GPCR) gene and gene sequences were analyzed.

**Results:**

Virus isolation on primary ovine fetal kidney and heart cell culture revealed a cytopathic effect at the third passage characterized by rounding of infected cells and margination of nuclear chromatin. Comparative sequence analysis of GPCR gene revealed that Egyptian LSDV isolated from Ismailia and Beni-Suef shared 99:100% nucleotide and amino acid (AA) identities with each other. In comparison to the vaccinal strains, Egyptian LSDV isolates shared 98:99 nucleotide and AA identities with LSDV Neethling vaccine strain and 93:94% with SPPV Romanian vaccine strain. No differences at the nucleotide or AAs were observed between the SPPV vaccine and virulent strains (100% identity). Phylogenetic analyses revealed that LSDV Neethling vaccine strain is more related to field Egyptian LSDV and clustered within the LSDV group while Romanian SPPV vaccine strain clustered in a separate clade with SPPV field isolates.

**Conclusion:**

Comparative sequencing and phylogenetic analyses of the GPCR gene reveal a minimal genetic variation between LSDV field isolates from different locations and a close relationship between virulent field strains and homologous vaccines.

## Introduction

Lumpy skin disease (LSD) and sheep pox are economically important *Capripoxvirus* (CaPV)-induced diseases of cattle and sheep, respectively. LSD virus (LSDV) and *Sheep pox virus* (SPPV) are categorized within the genus CaPV in the family *Poxviridae* [1].

LSD is an acute to subacute disease of cattle characterized by fever, rapid eruption of numerous circumscribed skin nodules, and generalized lymphadenitis [2-5]. The cost-effective of the disease was contributed to its high morbidity rate rather than mortality [5]. Geographically, the African continent was the first to record LSD incursions; however, the disease continues its spread to the Middle East and recently to Europe [1,5,6]. Regarding Egypt, the disease was first reported in May 1988 among cattle in Ismailia [7]. In early 2006, LSD reemerged in Egypt in a massive outbreak producing severe financial losses to livestock in different localities of Egypt [8].

Sheep pox is a highly contagious disease of small ruminants [9] characterized by fever, generalized pock lesions with high morbidity and mortality [4,10]. The disease regularly occurs in Asia and North Africa; later, several outbreaks were recorded in Greece [11] and Bulgaria [1].

There is no doubt that vaccination is the most effective way to control CaPV diseases [1]. Due to the cross-protection within the genus CaPV, any CaPV isolate could be used as a vaccine against LSDV [12]. In the endemic area with sheep pox, the live attenuated sheep pox vaccine was used for controlling both LSD and sheep pox.

In Egypt, the Kenyan SGP O-240 vaccine was used during LSD incursion in 2005-2006 [13]. At present, Romanian SPPV vaccine strain is used by Egyptian veterinary authorities to immunize both small ruminants and cattle against CaPVs; however, the reoccurrence of an outbreak in vaccinated animals has been reported [14]. Under this situation, molecular characterization of field virulent and vaccinal strains is necessary to determine their genetic relatedness.

In the current study, G protein-coupled receptors (GPCR) gene was used for molecular characterization and phylogenetic analysis of LSDV and SPPV circulating in the field along with vaccinal strains to assess their genetic relatedness.

## Materials and Methods

### Ethical approval

All animal handling procedures, as well as samples collection and disposal, were approved by the animal care and use committee of the Faculty of Veterinary Medicine, University of Sadat City, Egypt, according to the guidelines and recommendations of the European Communities Council Directive 1986 (86/609/EEC).

### Animals and clinical samples

From June 2015 to September 2016, LSD and sheep pox were suspected among dairy cattle herds located in private farms belonging to Ismailia (n=3) and Beni-Suef (n=2) Governorates and one sheep flock in Beni-Suef Governorate, respectively. All farms were clinically examined and a total of five nodular skin lesions were collected from diseased cattle with skin eruption all over the body while skin lesions were collected and pooled from infected sheep pox flock showing generalized pock lesions. The collected samples were kept at −20°C for virus isolation and polymerase chain reaction (PCR).

### Virus strains

South African Neethling vaccine strain (Lumpyvax®, MSD) each 1 mL (1 dose) of the vaccine contains 10^4^ TCID50 of freeze-dried, live, attenuated virus (SIS Neethling-type)from the Republic of South Africa and Romanian SPPV vaccine strain provided from the Pox Department, Veterinary Serum and Vaccine Research Institute, Abbassia, Cairo, Egypt, was used in the molecular study (10^3^ TCID50/ml).

### Virus isolation

Skin biopsies were used for the isolation of LSDV and SPPV according to Tuppurainen *et al*. [15]. Briefly, the skin tissue samples were minced and homogenized in a sterile condition by electric homogenizer (IKA, Model: T25D; Germany). A suspension of 10 mL of phosphate-buffered saline with antibiotics (0.1 mg gentamycin, 0.05 mg ampicillin, and 5 μg amphotericin B for each mL) was added, then left to stand overnight at 4°C. The suspension was centrifuged for 5 min at 2000 rpm to eliminate any gross particles, and then, 0.5 mL of supernatant was transferred into monolayer of primary ovine fetal kidney and heart cells growing in tissue culture flasks (T25) and maintained in Minimum Essential Medium, Hank’s salts with L-glutamine, 0.2% sodium bicarbonate, 10% fetal calf serum, 100 U/ml penicillin, and 100 µg/ml streptomycin. Cultures were observed daily for the cytopathic effect (CPE) for 14 days. Further, a passage for another 14 days was mandatory in case of negative culture into fresh monolayers.

### DNA extraction

DNA was extracted from skin lesions, infected cell culture, LSDV Neethling vaccine strain, and Romanian SPPV strain using a DNA/RNA Extraction Kit (Intron, Pathogen, Spin, cat#17154, Korea) according to the manufacturer’s instructions.

### PCR

The primers were developed to amplify the entire GPCR gene at position 6961-8119 according to Le Goff *et al*. [16]. The primers have the following gene sequences: 5-TTAAGTAAAGCATAACTCCAACAAAAATG-3´and 5´-TTTTTTTATTTTTTATCCAATGCTAATACT-3´). PCR was carried out in a total volume of 50 µl containing 25 µl master mix (Thermo Scientific, DreamTaq PCR Master Mix 2× Cat# K1071, USA), 1 µl of each primer (20 pmol), 5 µl of extracted DNA, and 18 µl of nuclease-free water. Neethling vaccine was used as control positive while Nuclease-Free water was used as control negative. The thermal profile of PCR was started with an initial denaturation at 94°C for 5 min and 35 cycles of denaturation at 94°C for 1 min, annealing at 50°C for 1 min, and extension at 72°C for 1 min, with a final extension of 7 min. The PCR products were visualized in transilluminator after being electrophoresed in 1.5% agarose gel.

### Sequences and phylogenetic analysis

PCR amplicons were purified using QIAquick PCR Purification Kit and dispatched to Macrogen™, Seoul, Korea, for DNA sequencing using two additional primers(5´-GATGAGTATTGATAGATACCTAGCTGTAGTT-3´and 5´-TGAGACAATCCAAACCACCAT-3´) according to Le Goff *et al*. [16]. BLAST analysis (http://Zwww.ncbi.nlm.nih.gov/blast) was initially implemented to establish sequence identity to GenBank accessions. Phylogenetic tree and sequence alignments were generated using MEGA(Molecular Evolutionary Genetics Analysis)Version X software. The tree was generated by the neighbor-joining method based on 1000 bootstrapped data sets [17].

## Results

### Clinical examination

**Figure-1:**
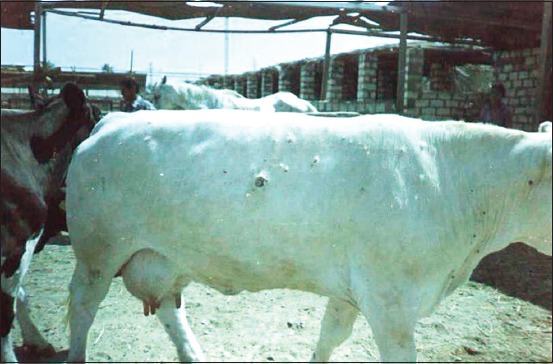
Skin lesions in a cow naturally infected with lumpy skin disease virus.

Fever, skin lesions scattered in different parts of the body (Figure-1), and enlargement of superficial lymph nodes were observed in examined cattle with or without edema in legs. Edema in brisket was also seen. Within a sheep flock, animals showed signs of cutaneous papules, especially in areas of skin without wool with nasocular discharge and fever.

### Virus isolation

**Figure-2:**
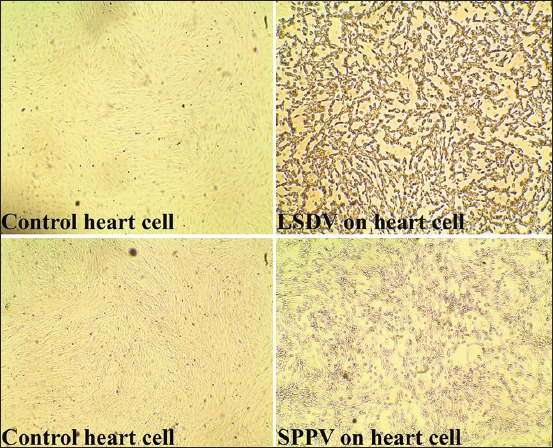
Lumpy skin disease virus and *Sheep pox virus* cytopathic at the third passage on cell culture.

Virus isolation revealed CPE at the third passage on primary ovine fetal kidney and heart cells, characterized by retraction of the cell membrane from surrounding cells, rounding of cells, and margination of the nuclear chromatin (Figure-2).

### PCR, gene sequencing, and sequence analyses

**Figure-3:**
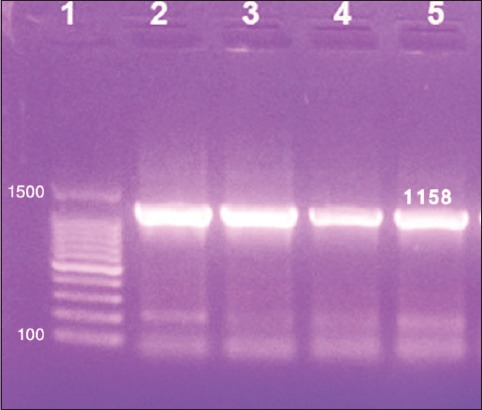
Gel electrophoresis of G protein-coupled chemokine receptor gene-based polymerase chain reaction. Lane 1: 100 bp DNA ladder, Lane 2: Lumpy skin disease virus (LSDV) (Neethling vaccine strain), Lane 3: *Sheep pox virus* (SPPV) (Romanian vaccine), Lane 4: LSDV (field strain), Lane 5: SPPV (field strain).

Using primer sequences targeting the entire GPCR gene, a fragment of 1158 bp has been amplified from all DNA extracts (Figure-3). Sequencing analyses of the GPCR gene revealed that five LSDV sequences obtained in the current study (MH427384.1, MH427385.1, MH427386.1, MH427387.1, and MH427388.1) are closely related to each other with nucleotide and amino acid (AA) identity ranged from 99% to 100% in between and 98:99% with LSDV Neethling vaccine. Comparative sequence analyses of GPCR gene reveal that field LSDV isolates differ from LSDV Neethling vaccine only in four AAs substitution (S/N76, M/I127, T/I268, and M/T328, respectively) and an AA deletion (Table-1) where AA at positions 30-33 was observed in MH427384.1, MH427385.1, MH427386.1, and MH427387.1 but not in LSDV Neethling vaccine.

**Table-1:**
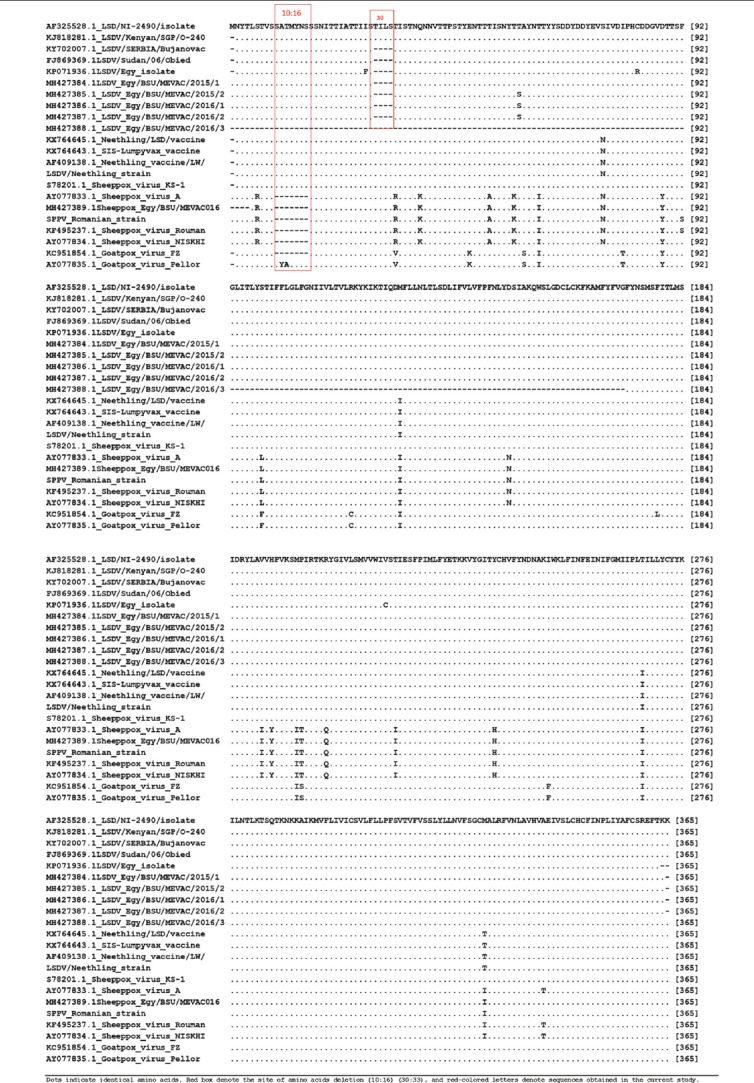
Deduced ammino acid sequence of G protein-coupled checmokine receptor gene.

**Table-2:** Differences in amino acid motifs between *Capripoxviruses* field and vaccine strains in G protein-coupled receptors gene.

Amino acid position	6	10	11	12	13	14	15	16	30	31	32	33	34	39	49	53	58	60
LSDV field (MH427384)	S	S	A	T	M	Y	N	S	-	-	-	-	T	Q	E	T	T	A
LSDV vaccine (KX764645)	.	.	.	.	.	.	.	.	T	I	L	S	.	.	.	.	.	.
SPPV field (MH427389)	R	-	-	-	-	-	-	-	T	I	L	S	R	K	.	A	K	.
SPPV vaccine (KF495237)	R	-	-	-	-	-	-	-	T	I	L	S	R	K	.	A	K	.
**Amino acid position**	63	76	80	88	99	117	127	149	191	193	198	199	204	218	238	249	268	328
LSDV field (MH427384: 88)	T	S	I	D	S	R	M	D	V	H	M	P	R	T	Y	I	T	M
LSDV vaccine (KX764645)	.	N	.	.	.	.	I	.	.	.	.	.	.	.	.	.	I	T
SPPV field (MH427389)	I	N	.	Y	L	.	I	N	I	Y	I	T	Q	I	H	.	I	T
SPPV vaccine (KF495237)	I	N	.	Y	L	.	I	N	I	Y	I	T	Q	I	H	.	I	T

Dashes and dots indicate missing amino acids and conserved residues at the corresponding positions, respectively. Blue boxes denote the eight unique profiles for LSDV, SPPV, and GTPV amino acids. Red boxes denote amino acid differences between LSDV and SPPV field strains. Green boxes denote amino acid differences between LSDV field and vaccine strains. LSDV=Lumpy skin disease virus, SPPV=*Sheep pox virus*, GTPV=Goat poxvirus

Regarding Romanian SPPV vaccine strain, it was found that AA residues situated in position 10-16 (SATMYNS) in LSDV field isolates are missed in SPPV vaccine; moreover, there are 27 variances in AA motifs between LSDV field isolates and SPPV vaccines were observed along GPCR gene (Tables-1 and 2). On the other hand, no differences between SPPV vaccinal and virulent strains were observed (100% nucleotide and AA identities).

**Figure-4:**
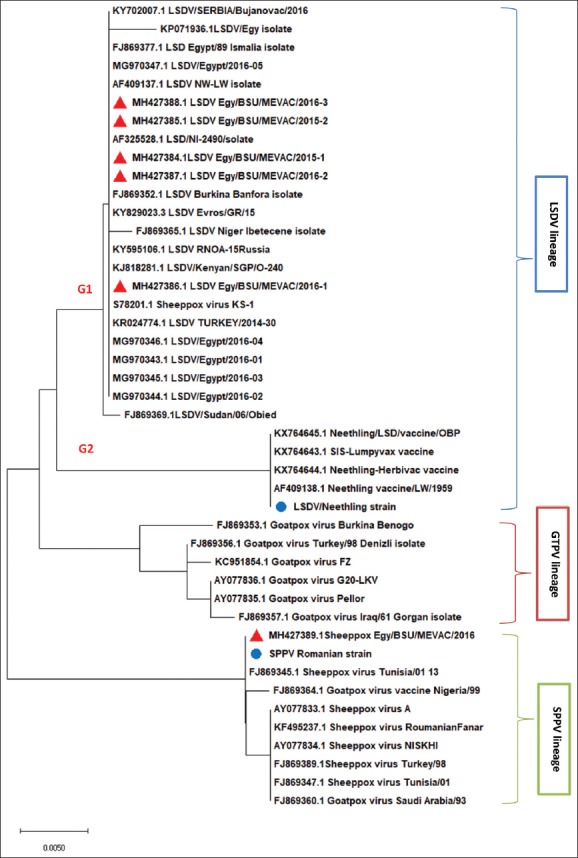
Phylogenetic analysis of the G protein-coupled chemokine receptor gene. The tree was generated using MEGA X program by the neighbor-joining analysis. Bootstrap confidence values were calculated on 1000 replicates according to the maximum likelihood approach. Sequences obtained in this study are labeled (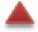 for lumpy skin disease virus and Sheep pox virus field isolates and 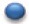 for vaccinal strains.

To represent the evolutionary relationships among field and vaccinal strains of LSDV and SPPV sequenced in this study and available CaPVs in the database, a GPCR-based phylogenetic tree was generated using the neighbor-joining method on nucleic acid sequences. The tree showed three tight genetic clusters (LSDV, goat poxvirus [GTPV], and SPPV lineages, respectively) (Figure-4). LSDV falls into two subgroups. Our LSDV field isolates were found in subgroup one with other virulent LSDV available in the database. The other subgroup comprised LSDV vaccinal strain including LSDV Neethling vaccine that was sequenced in the current study while Romanian SPPV vaccine strain was clustered in a separate clade with other virulent and vaccine strains of SPPV.

## Discussion

LSD and sheep pox diseases are now considered as endemic diseases in Egypt. Despite the extensive vaccination program adopted by Egyptian veterinary services, LSD and sheep pox are still prevalent and spread throughout the whole country, thereby the present study provides a molecular characterization of LSDV and SPPV recovered from field cases in Egypt and a comparison of *Capripoxviruses* field and vaccinal strains based on sequence analysis of GPCR gene.

A total of five LSDV and one SPPV were isolated from naturally infected animals with typical clinical features of LSD and sheep pox, respectively, after being confirmed by PCR.

The complete open reading frames of the GPCR gene of isolated viruses were sequenced along with vaccine strains. Comparative sequence analysis revealed that all of the five LSDV isolates are closely related to each other with a nucleotide and AA identity ranged from 99% to 100% in between confirming the circulation of the same virus strain.

Egyptian LSDV field isolates were found more related to LSDV Neethling vaccine where it differs only in four AA substitutions and an AA deletion at positions 30-33.

The comparative sequence analysis revealed that the 5-end of GPCR gene of SPPV vaccine was characterized by deletion of 21 nucleotides (7-aa) when it compared with LSDV field isolates. This sheep pox gap was recorded in all isolates and vaccine strains located in the database and was considered as a specific signature for SPPV as reported previously by Le Goff *et al*. [16]. Many variances in AA motifs between LSDV field isolates and SPPV vaccines were observed along GPCR gene. These variances include the eight unique AAs (S/R6, S/–10, A/–11, T/–12, T/R34, S/L99, P/T199, and M/I328, respectively) (Table-2) that are lineage-specific where AA signatures present either in LSDV or SPPV or GTPV as reported previously by Le Goff *et al*. [16] and El-Tholoth and El-Kenawy [18]. Interestingly, no differences between SPPV vaccinal and virulent strains were observed.

Phylogenetically, CaPV was delineated into three clades LSDV, GTPV, and SPPV as previously reported by Rouby *et al*. [19], Rouby [20], Rouby and Aboulsoud [21], and Mafirakureva *et al*. [22]. LSDV falls into two subgroups. LSDVs isolated in the current study were located in subgroup one with other virulent LSDV available in the database that proves the minimal genetic variation between different LSDV isolates from different locations and indicate the high stability of LSDVs as previously reported by Kara *et al*. [23]. The other subgroup comprised LSDV vaccinal strains including LSDV Neethling vaccine that was sequenced in the current study. Egyptian LSDV field isolates are more related to LSDV Neethling vaccine strain than to Romanian SPPV vaccine strain (currently authorized vaccine against LSD in Egypt). The high genetic relatedness between field LSDV isolates and LSDV Neethling vaccine strain was previously reported [23] and recent researches recommended the use of homologous vaccines for controlling CaPV-induced diseases combined with sufficient vaccination coverage and appropriate control measures [24].

## Conclusion

Comparative sequencing and phylogenetic analyses of GPCR gene revealed a minimal genetic variation between different LSDV isolates from different locations and a close relationship between LSDV Neethling vaccine strain and Egyptian field LSDV isolates. GPCR gene possesses specific signatures for LSDV and SPPV at both nucleotide and AA sequences level and cluster them separately according to their host origin.

## Authors’ Contributions

ME designed the study. SRR and AB performed PCR, sequence analysis, and wrote the initial draft of the manuscript. MW and ME revised the manuscript. All authors read and approved the final manuscript.
